# Multiple seborrheic keratoses in a previously irradiated site^⋆^^⋆⋆^

**DOI:** 10.1016/j.abd.2020.02.014

**Published:** 2020-08-16

**Authors:** Danielle Ferreira Chagas, Lúcia Martins Diniz, Bruna Anjos Badaró, Elton Almeida Lucas

**Affiliations:** Dermatology Service, Hospital Universitário Cassiano Antônio Moraes, Vitória, ES, Brazil

Dear Editor,

Breast cancer is the second most common malignancy worldwide. In Brazil, 59,700 new cases were estimated for 2019. It predominantly affects women in their 50s, and the most common histological subtype is invasive ductal adenocarcinoma.^1^

Treatment is associated with dermatological complaints in 74%–100% of cases.^2^ Therapy involves surgery and locoregional radiotherapy, in addition to chemotherapy and hormone therapy for systemic treatment.^3^

Seborrheic keratosis is the most common benign cutaneous tumor, being predominantly observed in Caucasian adults. It originates in the epidermis, and a proliferation of immature keratinocytes is observed. Clinically, it is characterized by brownish, well-defined papules with agreasy surface.^4^, ^5^ Based on clinical suspicion, dermoscopy helps in its diagnosis, which is confirmed by histopathology.^5^

A case of multiple seborrheic keratoses restricted to the site of previous breast cancer radiotherapy is described.

A female patient, 73 years old, white, was diagnosed four years ago with invasive micropapillary carcinoma in the left breast, which required surgical intervention (quadrantectomy), chemotherapy (adriamycin, cyclophosphamide, and paclitaxel) and 30 adjuvant radiotherapy sessions. Six months ago, she noticed the onset of brownish, asymptomatic, slowly evolving papules, restricted to the skin adjacent to the surgical scar, an area previously irradiated (Figure 1, Figure 2). In 2019, she sought dermatological care, and the clinical examination and dermoscopy showed lesions suggestive of seborrheic keratoses, on the left breast only. At the moment, the patient is in remission and is being followed-up every six months. The histopathology of a lesion was compatible with the diagnosis, due to the presence of basaloid cells, hyperkeratosis, and formation of horn pseudocysts (Fig. 3).

**Figure 1:**
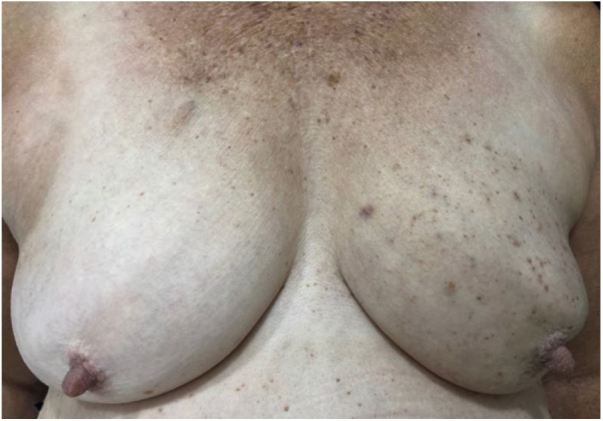
Multiple seborrheic keratoses restricted to the previously irradiated site.

**Figure 2:**
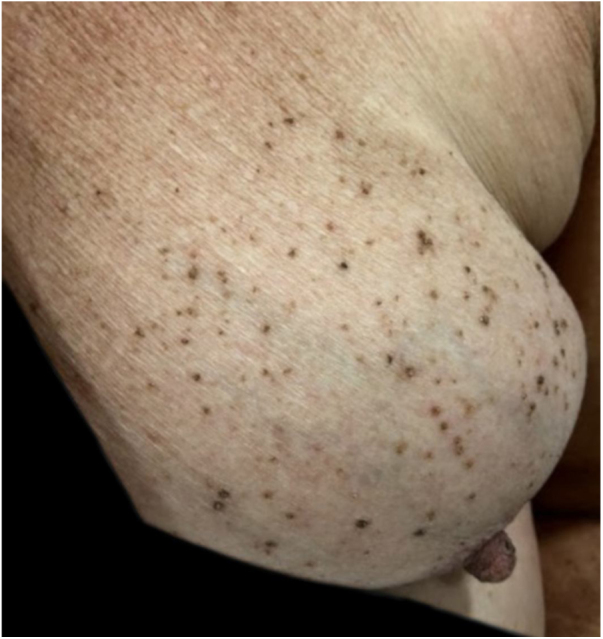
Detail of multiple brownish, rounded, well-defined papules with a greasy surface, restricted to the left breast.

**Figure 3:**
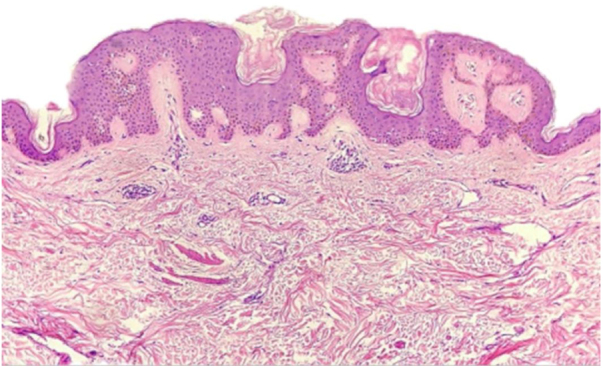
Histopathological examination of one of the breast lesions, showing proliferation of uniform basaloid cells, elongation of the rete ridges, and hyperkeratosis. Keratin pseudocysts and melanic hyperpigmentation of the epidermis are also observed (Hematoxylin & eosin, ×10).

By 2015, over 1.3 million people had been diagnosed with breast cancer worldwide.^1^ The invasive micropapillary subtype, diagnosed in this case, is rare, representing 0.9%–2% of breast carcinomas; it is associated with aggressive clinicopathological characteristics.^1^

Radiotherapy has numerous effects on skin tissue, ranging from acute (up to six months after therapy initiation) to chronic; depending on the location, size, and depth of the irradiated tumor, these effects can be classified as mild, moderate, or severe.^2^, ^3^, ^5^ The patient presented chronic symptoms.

The severity of the skin lesions caused by radiation is dependent on factors related to the treatment and the patient.^2^ Those linked to treatment include the total dose and the irradiation site, fractionation time, volume and area of irradiated tissue, and the use of chemotherapy.^2^, ^3^ Among the patient-related factors, smoking, malnutrition, obesity, autoimmune diseases, and genetic factors are noteworthy.^2^, ^3^ The only risk factors for the present patient were multiple radiotherapy and chemotherapy sessions.

The sign of Leser-Trélat is rare, and characterized by the abrupt eruption of multiple seborrheic keratoses, usually on the back, which may precede or occur after the diagnosis of the malignancy, especially lung and gastrointestinal adenocarcinoma.^4^ The patient presented progressive lesions only at the irradiated site.

The mechanism of lesion determined by radiation is not fully understood; however, it is known that the skin is an organ in constant renewal, consisting of cells with rapid proliferation and maturation, making them vulnerable to this therapy.^2^ Basal keratinocytes and hair follicles are highly radiosensitive. Moreover, radiation damage causes inflammation, cell recruitment, DNA damage, and cytokine generation.^3^, ^5^

The etiology of seborrheic keratosis remains unknown. Recently, it has been postulated that epidermal growth factors (oncogenes [PIK3CA] and fibroblast growth factor receptor 3 [FIGR3]) may participate in 32% and 48% of cases, respectively. Recent studies demonstrated that other oncogenes (TERT and DPH3) are also involved in the genesis of the lesions.^5^

Eczemas and chemotherapy can trigger or increase inflammation in pre-existing seborrheic keratoses, differently from the case presented.^3^

The diagnosis of seborrheic keratosis is clinical, and dermoscopy is useful for differentiating it from other pigmented lesions. The dermoscopic pattern is polymorphic; the most characteristic finding is horn pseudocysts, as observed in the present case. In case of diagnostic doubt, histopathology becomes indispensable.^5^ In our patient, due to the presence of multiple lesions, the authors decided to excise one of them for histopathological examination, confirming the clinical diagnosis.

Treatment is indicated for aesthetic purposes; some options include curettage, application of trichloroacetic acid, and cryotherapy with liquid nitrogen.^5^ In the present case, cryotherapy was applied in two sessions; a reduction in the greasy surface of seborrheic keratoses and hyperpigmentation was observed in the first session.

In the literature review, no studies on seborrheic keratosis induced by radiotherapy were retrieved; the authors concluded that radiation triggered the proliferation of keratinocytes, probably through mediators that lead to the production of melanogenesis-stimulating cytokines, activation of oncogenes, and epidermal growth factors.

## Financial support

None declared.

## Authors’ contributions

Danielle Ferreira Chagas:Drafting and editing of the manuscript; intellectual participation in the propaedeutic and/or therapeutic conduct of the studied cases; critical review of the literature.

Lúcia Martins Diniz: Approval of the final version of the manuscript; intellectual participation in the propaedeutic and/or therapeutic conduct of the studied cases; critical review of the manuscript.

Bruna Anjos Badaro:Intellectual participation in the propaedeutic and/or therapeutic conduct of the studied cases.

Elton Almeida Lucas:Intellectual participation in the propaedeutic and/or therapeutic conduct of the studied cases.

## Conflicts of interest

None declared.
